# Effect of the Addition of Polyacrylic Acid of Different Molecular Weights to Coagulation Bath on the Structure and Performance of Polysulfone Ultrafiltration Membranes

**DOI:** 10.3390/polym15071664

**Published:** 2023-03-27

**Authors:** Tatiana Plisko, Katsiaryna Burts, Anastasia Penkova, Mariia Dmitrenko, Anna Kuzminova, Sergey Ermakov, Alexandr Bildyukevich

**Affiliations:** 1 St. Petersburg State University, 7/9 Universitetskaya nab., 199034 St. Petersburg, Russia; 2 Institute of Physical Organic Chemistry, National Academy of Sciences of Belarus, 220072 Minsk, Belarus

**Keywords:** ultrafiltration, fouling, polysulfone, polyacrylic acid, molecular weight, hydrophilization, membrane modification

## Abstract

Membrane fouling is a serious issue in membrane technology which cannot be completely avoided but can be diminished. The perspective technique of membrane modification is the introduction of hydrophilic polymers or polyelectrolytes into the coagulation bath during membrane preparation via non-solvent-induced phase separation. The influence of polyacrylic acid (PAA) molecular weight (100,000, 250,000 and 450,000 g·mol^−1^) added to the aqueous coagulation bath (0.4–2.0 wt.%) on the polysulfone membrane structure, surface roughness, water contact angle and zeta potential of the selective layer, as well as the separation and antifouling performance, was systematically studied. It was found that membranes obtained via the addition of PAA with higher molecular weight feature smaller pore size and porosity, extremely high hydrophilicity and higher values of negative charge of membrane surface. It was shown that the increase in PAA concentration from 0.4 wt.% to 2.0 wt.% for all studied PAA molecular weights yielded a substantial decrease in water contact angle compared with the reference membrane (65 ± 2°) (from 27 ± 2° to 17 ± 2° for PAA with M_n_ = 100,000 g·mol^−1^; from 25 ± 2° to 16 ± 2° for PAA with M_n_ = 250,000 g·mol^−1^; and from 19 ± 2° to 10 ± 2° for PAA with M_n_ = 450,000 g·mol^−1^). An increase in PAA molecular weight from 100,000 to 450,000 g·mol^−1^ led to a decrease in membrane permeability, an increase in rejection and tailoring excellent antifouling performance in the ultrafiltration of humic acid solutions. The fouling recovery ratio increased from 73% for the reference membrane up to 91%, 100% and 136% for membranes modified with the addition to the coagulation bath of 1.5 wt.% of PAA with molecular weights of 100,000 g·mol^−1^, 250,000 g·mol^−1^ and 450,000 g·mol^−1^, respectively. Overall, the addition of PAA of different molecular weights to the coagulation bath is an efficient tool to adjust membrane separation and antifouling properties for different separation tasks.

## 1. Introduction

Membrane fouling is a serious issue in membrane technology which cannot be completely avoided but can be diminished [1]. Membrane fouling is caused by the sorption and upbuilding of biomolecules, solid or colloid particles dissolved in feed mixture on the surface and inside pores of the membrane selective layer due to concentration polarization [2,3]. Membrane fouling leads to the degradation of membrane performance, decrease in permeate purity, contamination of the membrane modules, permeation by microorganisms and decrease in membrane lifespan and, as a result, a substantial rise in membrane separation costs [1,2,3,4]. Membrane fouling causes a decrease in membrane flux when the transmembrane pressure is kept constant in the system or the necessity to increase transmembrane pressure when the flux has to be kept constant [4]. Mitigation of membrane fouling always yields significant economic benefits. Therefore, enormous efforts in membrane research are devoted to solving this problem [5,6].

There are several approaches to mitigate membrane fouling, including modification of the membrane surface, pretreatment of the feed media, membrane cleaning and adjusting operation conditions of the membrane plant [7,8]. In fact, only simultaneous application of all these approaches can efficiently reduce membrane fouling in industry. It is worth noting that fouling mitigation requires additional investments into the equipment for cleaning and feed pretreatment, as well as additional place for storage and handling of cleaning reagents. Moreover, the necessity of cleaning yields interruptions in the membrane separation and reduces productivity of the membrane plant. One of the critical issues in fouling mitigation is the interaction of the membrane surface with the foulants in the feed mixture upon filtration [9,10,11,12]. Tuning this interaction can significantly reduce adsorption of foulants on the membrane surface [9]. The construction of antifouling membrane surfaces usually includes manipulating membrane surface chemistry, pore size, hydrophilic–hydrophobic balance, wettability, surface roughness and charge [10,11,12].

The following techniques were applied for membrane modification to decrease the degree of membrane fouling:

(1) Bulk modification by blending hydrophilic additive to the casting solution;
(2) Surface grafting of polymers and hydrophilic molecules;
(3) Plasma treatment;
(4) Surface coating;
(5) Introduction of hydrophilic polymers into the coagulation bath during membrane preparation [13,14].

Bulk modification by blending hydrophilic polymers to the casting solution is a widely used technique to increase the hydrophilicity of the membrane surface. Polyethylene glycol (PEG) [15], polyvinylpyrrolidone (PVP) [16,17], block copolymers of polyethylene glycol and polypropylene glycol (Pluronic^®^, Synperonic, Tetronic) are the most suitable for this purpose [18,19,20,21,22,23]. One of the disadvantages of this technique is the leaching out of hydrophilic polymer during membrane preparation via non-solvent-induced phase separation (NIPS) due to their high hydrophilicity and solubility in water (the most frequently used coagulant).

Membrane modification by surface grafting of hydrophilic polymers to the surface of the membrane selective layer results in the formation of a hydrophilic steric barrier that reduces absorption of colloids, solid particles or proteins [24,25,26,27]. Surface grafting is a flexible technique which allows tailoring desirable surface properties by application of various monomers and adjusting the locations of grafted substances on the membrane surface. However, the application of additional monomers and solvents in the membrane modification process increases the cost of membrane production and negative environmental impact [24,25,26,27]. Moreover, the additional step of membrane modification is time- and labor-consuming [24,25,26,27].

Membrane modification by plasma treatment is implemented using ionized gas generated by a strong electrical discharge in a low-pressure area. Such an attack on the membrane surface causes homolytic bond breakage that forms free radicals that are capable of reacting with other molecules placed close to the polymer surface. Plasma surface treatment can be accomplished using different gases (e.g., carbon dioxide, helium, hydrogen, argon, nitrogen and oxygen). The main goals of plasma treatment are (1) direct surface functionalization (generation of highly hydrophilic hydroxyl, carboxylic, carbonyl and amine groups); (2) formation of surface active groups (for instance peroxides), which are used for grafting of polymers or graft polymerization; and (3) changing surface topology, ξ-potential and hydrophilicity. The disadvantages of this approach are possible damage of the membrane surface, which can deteriorate membrane performance, and the need for expensive equipment and thus difficulties in scaling up this method [28,29,30,31,32].

Membrane surface coating usually involves immersing the surface into the modification solution, followed by the removal of residual solvents. In this case, solutions of polyvinyl alcohol (PVA), PEG, PVP, etc., are widely used [13,33,34,35,36]. The Langmuir–Blodgett technique, physical adsorption of surfactants, polymers or monomers with the subsequent polymerization and layer-by-layer deposition of polyelectrolytes can be applied [13,33,34,35,36]. Coating allows applying this type of modification to the commercial membrane modules of any configuration using static or dynamic adsorption techniques. It should be mentioned that coating does not alter the internal membrane structure, nor influence mechanical strength, and it does not require expensive equipment. However, sometimes it demands complex preparation of modification solutions, their reflux and homogenization and other treatment procedures [36]. 

The perspective technique of membrane modification is the introduction of hydrophilic polymers or polyelectrolytes into the coagulation bath via membrane preparation. It mostly refers to membrane preparation via NIPS. This technique can contribute to surface modification due to the embedding of hydrophilic polymers or polyelectrolytes into the selective layer, which yields changes in the hydrophilicity, roughness, charge and chemical composition of the membranes. This approach was developed and studied in [37,38,39,40,41,42,43,44,45,46,47,48,49,50,51,52,53,54,55]. 

Polyacrylic acid (PAA) [48,49,53], polyethylene imine (PEI) [37,41,43], copolymers Praestol 859 [47,51] and Praestol 2540 [50], poly (diallyldimethylammonium chloride) [41], chitosan [55], zwitterionic copolymers [56], poly (sodium 4-styrenesulfonate) (PSS) [40], PVP [52] and PVA [54] were applied as additives to the coagulant for preparation of flat-sheet and hollow fiber membranes. There are different purposes which can be achieved by the addition of hydrophilic polymers and polyelectrolytes to precipitation medium:

(1) Increase in membrane antifouling performance [47,48,49,50,51,52,53,54,56];
(2) Formation of a separation layer after subsequent cross-linking [37,38] or reaction with oppositely charged polyelectrolyte [37,40,41,42,45,46];
(3) Creating an intermediate layer for preparation of the composite membrane via interfacial polymerization (IP) technique [44];
(4) Tailoring membrane surface charge for enhanced separation of charged molecules [39].

Previously, Burts et al. [48,49] and Bildyukevich et al. [53] studied the surface modification of polysulfone (PSF) [48,49] and polyethersulfone (PES) membranes [53] by addition of PAA with a molecular weight of 250 kDa to the coagulant. It was shown that using PAA aqueous solutions as coagulation bath decreased the coagulation rate due to the rise in precipitation medium viscosity and abatement of the mutual diffusion of solvent and coagulant, which yielded a decrease in the pore size of the membrane selective layer [48,49]. The addition of PAA to the coagulation bath led to a decline in membrane pure water flux and a rise in PVP (Mw = 40,000 g·mol^−1^) and lysozyme rejections, as well as a substantial increase in resistance to fouling in the filtration of humic acid solution and thermomechanical pulp mill process water [48,49]. Moreover, membranes prepared using aqueous solutions of PAA as coagulation medium demonstrated higher flux and hemicelluloses retention in thermomechanical pulp mill process water treatment, which allowed purifying hemicelluloses from lignin [48,49,53]. 

In [54], an aqueous solution of PVA was applied as a surface hydrophilizing agent for polyvinylidene fluoride (PVDF) ultrafiltration membrane modification during its preparation via the NIPS technique. It was revealed that membrane precipitation in the PVA aqueous solution led to the enhanced surface hydrophilicity manifested in the water contact angle decrease from 72° to 53°. Moreover, it resulted in pure water flux decline from 130 L·m^−2^·h^−1^ (water coagulation bath) to 14 L·m^−2^·h^−1^ (1.0 wt.% PVA aqueous solution as coagulation bath), as well as in the enhancement of membrane resistance to fouling.

To prepare composite hollow fiber membranes for nanofiltration [37,38,40,41,42,45] and forward osmosis [46] in one step, a “chemistry in a spinneret” method proposed by Kopec et al. [39] was applied and further developed. According to this method, membrane-forming polymer or an additive in the dope solution reacts with a monomer/crosslinker of polyelectrolyte added to the bore coagulant upon hollow fiber membrane spinning. It yields the formation of a composite hollow fiber membrane via a single step. This approach paves the route to commercial production of thin-film composite hollow fiber membranes, which are not scaled-up yet due to the difficulties in creating a defect-free layer on the surface with complex geometry. For instance, PES dope solution comprising trimesoyl chloride (TMC) as a cross-linker and a bore fluid with PEI additive was used to obtain a nanofiltration membrane which demonstrated a molecular weight cut-off (MWCO) of 1 kDa [38]. Gherasim et al. added PSS to the dope solution and PEI or PDADMAC to the bore fluid to prepare dual-charged hollow fiber membranes with an MWCO of 0.3 kDa [57]. Gao et al. introduced sulfonated polysulfone in the PES dope solution and added PEI in the internal coagulant. The amine groups were successfully immobilized on the selective layer surface of hollow fiber membranes, which allowed the further subsequent cross-linking of the separation layer by PAA, glutaraldehyde (GA) or TMC. As a result, nanofiltration membranes for heavy metal removal with an MWCO of 0.157 kDa were developed [37]. Emonds et al. combined the formation of polyelectrolyte complex and covalent cross-linking: PEI was added to the PES dope solution and PSS and GA were added to the internal coagulant during preparation of hollow fiber membranes, which yielded the development of hollow fiber membranes with high salt rejection, high mechanical stability and an MWCO of 0.35 kDa [40].

The addition of polyelectrolytes to the bore fluid can be used to create an intermediate layer for preparation of thin-film hollow fiber membranes for reverse osmosis via IP technique [44]. For instance, Mohammadifakhr et al. increased the success rate of the formation of a defect-free polyamide layer up to 86–100% (compared with 29% for reference membranes without intermediate layer) on the inner surface of hollow fiber membranes by formation a polyelectrolyte complex between PSS added to the dope solution and either PEI or PDADMAC added to the bore fluid [44]. 

Introduction of polyelectrolyte (sulphonated poly(ether ether ketone)) to the bore fluid during hollow fiber membrane spinning was applied to tailor pH-dependable charge properties to the selective layer of the polyimide membrane. It allowed enhancing the separation of bovine serum albumin and hemoglobin, which are of similar molecular weight but different isoelectric point and charge. It was reported that modification with the addition of sulphonated poly(ether ether ketone) to the bore fluid resulted in enhanced selectivity (around 200) of bovine serum albumin and hemoglobin in their binary mixture at pH = 7.5 [39].

Nevertheless, not only the nature and concentration of hydrophilic polymer or polyelectrolyte additive in the precipitation medium but also its molecular weight are important and affect the structure and performance of membranes. However, a literature review showed that there were not any studies concerning the effect of molecular weight of the polyelectrolyte as an additive to the coagulation bath during membrane preparation via NIPS on the membrane hydrophilic–hydrophobic properties, structure, performance and antifouling stability. Based on this, in the present paper, the effect of the PAA molecular weight (100,000, 250,000 and 450,000 g·mol^−1^) and concentration added to the coagulation bath during membrane preparation via NIPS was investigated for the first time. This study is a continuation of the previous systematic research on the influence of the addition of polyelectrolytes to the coagulation bath during membrane preparation via NIPS [47,48,49]. The influence of PAA molecular weight on the polysulfone membrane structure, surface topography, hydrophilic–hydrophobic properties and zeta potential of the selective layer, as well as the separation and antifouling performance, were systematically studied. 

## 2. Materials and Methods

### 2.1. Materials 

Ultrafiltration membranes were prepared via NIPS from casting solutions containing polysulfone (PSF, Ultrason S 6010, *M_n_* = 55,000 g·mol^−1^, BASF) with the addition of polyethylene glycol (*M_n_* = 400 g·mol^−1^, PEG-400, BASF) in *N*,*N*-dimethylacetamide (DMAc, 99%, BASF). Polyacrylic acid (PAA, Sigma-Aldrich, St. Louis, MO, USA) of three molecular weights (100,000, 250,000 and 450,000 g·mol^−1^) was applied as an additive to precipitation bath. PAA concentration was varied from 0 to 2.0 wt.%. 

Polyvinylpyrrolidone (PVP K-30, *M_n_* = 40,000 g·mol^−1^, Sigma-Aldrich, St. Louis, MO, USA) and lysozyme (*M_n_* = 14,300 g·mol^−1^, pI = 11.35, Sigma-Aldrich, St. Louis, MO, USA) were applied as test substances to determine membrane separation properties. The solution of fertilizer (Hydrohumin, Biochem, Svisloch, Belarus) in tap water was used as a source of humic acids and applied for membrane separation and antifouling performance studies. The content of humic acids in the aqueous solution was 0.005 wt.%.

### 2.2. Methods

#### 2.2.1. Preparation of PSF Ultrafiltration Membranes

Asymmetric porous membranes were obtained using casting solution containing 20 wt.% PSF, 10 wt.% of PEG-400 and 70 wt.% of DMAc by standard procedure reported in [48,49]. Casting solution was obtained by mixing all components at 100 °C for 5 h using an overhead stirrer at 1000 rpm. The solution was cast on a glass plate with the use of a casting blade with a gap height of 250 μm. Water and 0.4–2.0 wt.% PAA aqueous solutions of different molecular weights (100,000, 250,000 and 450,000 g·mol^−1^) were applied as nonsolvents in membrane preparation via NIPS. Membranes were left in the distilled water for 24 h for removing residual solvent. Membrane abbreviations are listed in Table 1. 

#### 2.2.2. Investigation of Membrane Separation Performance 

Membrane pure water flux (PWF) (J, L·m^−2^·h^−1^) was studied at the temperature of 20 °C and transmembrane pressure of 0.1 MPa. Rejection (%) of membranes was investigated by ultrafiltration of aqueous PVP K-30 solution (0.3 wt.%) and lysozyme solution in the phosphate buffer (0.5 wt.%) (pH 7.2–7.4 ± 0.05). The procedure of the measurement of transport properties was previously published in [20]. 

The PVP K-30 rejection was measured with interferometer LIR-2 (Zagorsk Optical and Mechanical Plant, Sergiyev Posad, Russia). The lysozyme rejection was analyzed using spectrophotometer Metertech SP 8001 (Metertech Inc., Taipei, Taiwan) at a wavelength of 280 nm. Parameter deviations did not exceed 0.5%. To calculate the rejection (R, %), Equation (1) was applied:

(1) $$R=\left(1-\frac{C_{p}}{C_{f}}\right)·100$$

where C_p_ and C_f_ are the concentrations of the test substance in permeate and feed solution, correspondingly.

#### 2.2.3. Study of Membrane Antifouling Performance

The investigation of membrane antifouling performance was carried out using 0.005 wt.% HAs solution in tap water. Firstly, distilled water was filtered for 30 min at 0.1 MPa to reach the steady state flux of the ultrafiltration membrane, and thereafter, the PWF was measured. HAs solution was filtered for 1 h at 0.15 MPa, and flux was determined every 15 min. After that, the HAs solution was replaced by distilled water in the ultrafiltration cell, and the membranes were cleaned by filtering the distilled water for 30 min at 0.1 MPa. The pure water flux of the cleaned membranes (*J_c_*) was determined.

This protocol was iterated two times. The flux recovery ratio (FRR) after filtration and the total flux decline ratio (DT) were determined by Equations (2) and (3), respectively:

(2) $$FRR=\left(\frac{J_{c}}{PWF}\right)\cdot 100\%$$

(3) $$DT=\left(\frac{PWF-J_{p}}{PWF}\right)\cdot 100\%$$

where *J_c_* is the pure water flux of the membrane after cleaning with distilled water, L m^−2^ h^−1^; *J_p_* is the flux of HAs solution, L m^−2^ h^−1^.

The spectrophotometer Metertech SP 8001 (Metertech Inc., Taipei, Taiwan) was applied for determination of the optical density of HAs feed solution and permeate at a wavelength of 400 nm. Concentration of iron was analyzed by an inductively coupled plasma atomic emission spectrometer (Vista PRO, Simi Valley, CA, USA). 

#### 2.2.4. Investigation of the Composition of Membranes

The studies of the skin and bottom layer compositions of membranes were carried out using Fourier transform infrared (FTIR) spectroscopy by spectrometer Nicolet Is50 (Thermofisher Scientific, Waltham, MA, USA) in the wavelength range of 400–4000 cm^−1^ at 25 °C. To prepare membrane samples, they were dried at room temperature for 5 days. 

#### 2.2.5. Study of Membrane Structure

The structure of the membrane selective layer was investigated by scanning electron microscope (SEM) Zeiss Merlin (Carl Zeiss AG, Oberkochen, Germany). The differences in the membrane cross-sections’ structures after modification by PAA were studied by SEM Phenom Pro (Thermofisher Scientific, Waltham, MA, USA). Samples of membrane cross-sections were prepared by splitting in liquid nitrogen followed by the covering of the gold layer using DSR1 coater (Vaccoat, London, UK). 

#### 2.2.6. Study of the Topography of Membrane Skin Layer

Membrane surface topography was studied by atomic force microscope (AFM) NT MDT nTegra Maximus (NT-MDT Spectrum Instruments, Zelenograd, Russia).

#### 2.2.7. Investigation of ξ-Potential of Membrane Skin Layer

ξ-potential of the skin layer of PSF and PSF/PAA membranes was evaluated using electrokinetic analyzer SurPASS 2 (Anton Paar, Graz, Austria). The protocol of measurements was described in [47,48]. The dependencies of the ξ-potential on the pH in the range of 3–10 were obtained.

#### 2.2.8. Measurement of Water Contact Angle of the Selective Layer

The water contact angle of the membrane skin layer was investigated by the attached bubble method via the spontaneous placement of the air bubble on the membrane surface immersed in the distilled water using an instrument LK1 (“Open Science”, Krasnodar, Russia). The procedure is described previously in detail [48]. Deviations of water contact angle values did not exceed 2°.

## 3. Results

Previously, it was shown that the introduction of PAA (M_w_ = 250,000 g·mol^−1^) to the coagulation bath upon formation of flat sheet membranes from PSF and PES via NIPS yielded the incorporation of PAA macromolecules into the membrane skin layer but not to the underside layer [48,49,53]. Due to precipitation of membrane-forming polymer in the medium of PAA aqueous solution, the speed of exchange of solvent and nonsolvent in NIPS decreased since the coagulant viscosity increased. It led to the formation of the skin layer with higher thickness and density, smaller size of pores and lower porosity. This study aims to reveal the influence of PAA molecular weight added to coagulation medium on the morphology and transport properties of PSF ultrafiltration membranes.

### 3.1. Influence of the PAA Molecular Weight on the Membrane Composition

**Figure 1:**
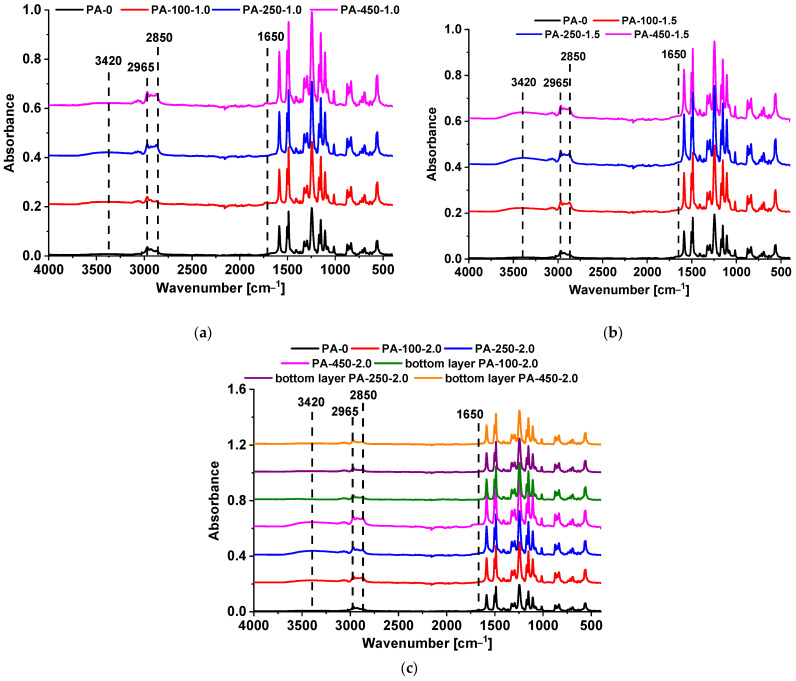
FTIR spectra of the skin and underside layers of PSF membranes obtained using PAA of different molecular weights as an additive to the coagulation medium. PAA concentration, wt.%: (**a**) 1.0; (**b**) 1.5; and (**c**) 2.0.

FTIR spectra of the skin and underside layer surfaces are shown in Figure 1 and Figure S1 (Supplementary Materials). Figure 1 presents the differences in FTIR spectra depending on PAA molecular weight at 1.0 wt.%, 1.5 wt.% and 2.0 wt.% PAA in the precipitation medium. Figure S1 shows the comparison of FTIR spectra of the membranes with various PAA concentrations in the precipitation bath within one PAA molecular weight (100,000, 250,000 or 450,000 g·mol^−1^).

Similarly to [47,48], it can be seen that vibrations of the main membrane material PSF (1153, 1583, 1487, 1106, 1243, 2850 and 2965 cm^−1^) predominate in all spectra (Figure 1 and Figure S1). The immobilization of PAA macromolecules can be proved only by the peak at 1650 cm^−1^ assigned to the oscillations of C=O in carboxylic groups of PAA and broad absorption band at 3000–3600 cm^−1^, with the maximum at 3420 cm^−1^, which is attributed to the stretching of O–H groups associated by the hydrogen bond (Figure 1 and Figure S1). The small amount of immobilized PAA in the skin layer in comparison with the amount of PSF is the reason for the low intensity of the vibrations attributed to PAA.

According to the FTIR spectra, the immobilization of PAA occurred mainly on the skin layer surface and not on the bottom layer of membranes, even at the highest studied PAA concentration (2.0 wt.%) (Figure 1c). As it was discussed earlier [47,48,49,50,51], the immobilization of PAA predominantly in the skin is explained by the following:

(1) Absence of the contact of the membrane bottom layer with the precipitation medium until the formation and detachment of the flat-sheet membrane from the glass plate;
(2) Low rate of diffusion of PAA macromolecules inside partly precipitated polymer film during NIPS.

The intensity of the PAA oscillations was found to increase with the increase in PAA molecular weight at all studied concentrations (Figure 1), as well as with the increase in PAA concentration (Figure S1). When the molecular weight of PAA rises (and, hence, the length of the macromolecular chain of PAA), more physical contacts can be formed between PAA and PSF. So, PAA is more firmly anchored to the membrane matrix. Moreover, PAA macromolecules with higher molecular weight fixed in the selective layer have more monomer units (and, hence, more carboxylic groups), which yield higher band intensity for these groups in FTIR spectra. When PAA molecular weight increases, the possibility of formation of PAA associates due to the formation of intermolecular hydrogen bonds in the aqueous solution rises. This can facilitate more PAA macromolecules to be embedded into the skin layer, since they are entangled in the associate.

Thus, it is expected that addition of PAA with the molecular weight of 450,000 g·mol^−1^ to the coagulation medium will have a more significant effect on the properties of membrane skin layer compared with PAA with 250,000 and 100,000 g·mol^−1^ at similar contents, because more carboxylic groups are immobilized on the membrane surface.

### 3.2. Influence of the PAA Molecular Weight and Concentration in Coagulation Medium on the Membrane Structure

**Figure 2:**
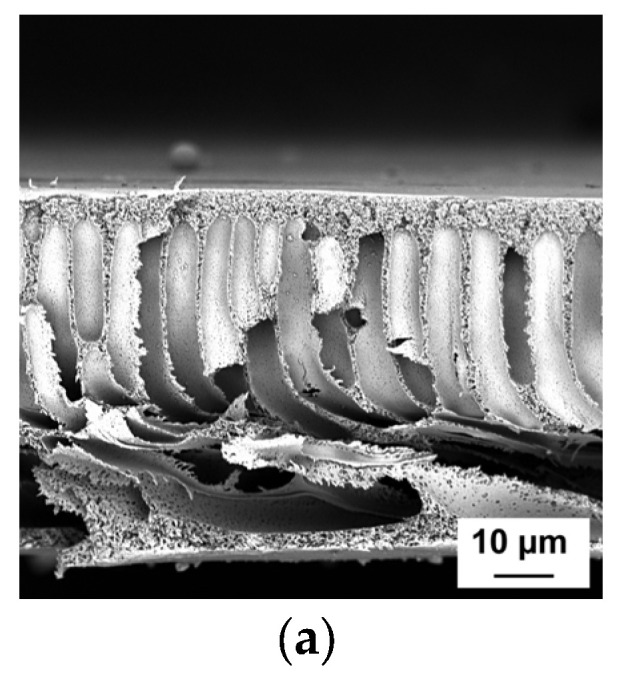

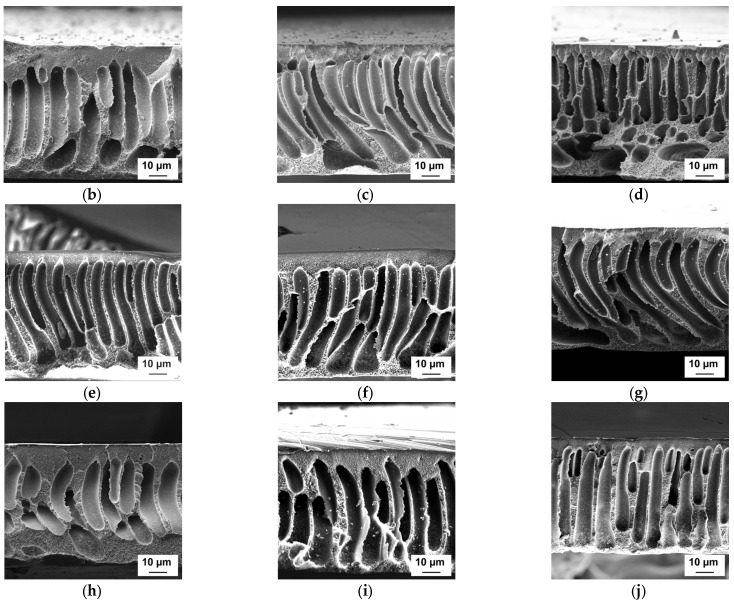
SEM micrographs of the cross-sections of the reference PSF membrane (**a**) and membranes, modified by the introduction of PAA to the precipitation bath (**b**–**j**): (**a**) PA-0; (**b**) PA-100-1.0; (**c**) PA-250-1.0; (**d**) PA-450-1.0; (**e**) PA-100-1.5; (**f**) PA-250-1.5; (**g**) PA-450-1.5; (**h**) PA-100-2.0; (**i**) PA-250-2.0; and (**j**) PA-450-2.0.

**Figure 3:**
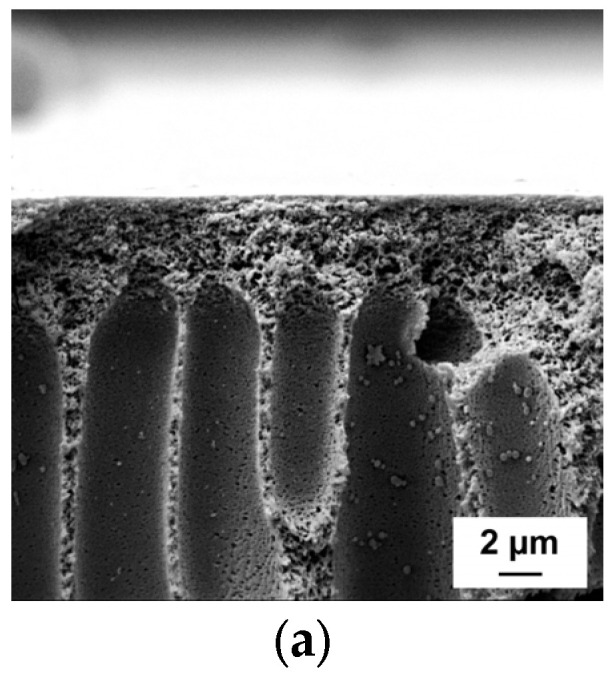

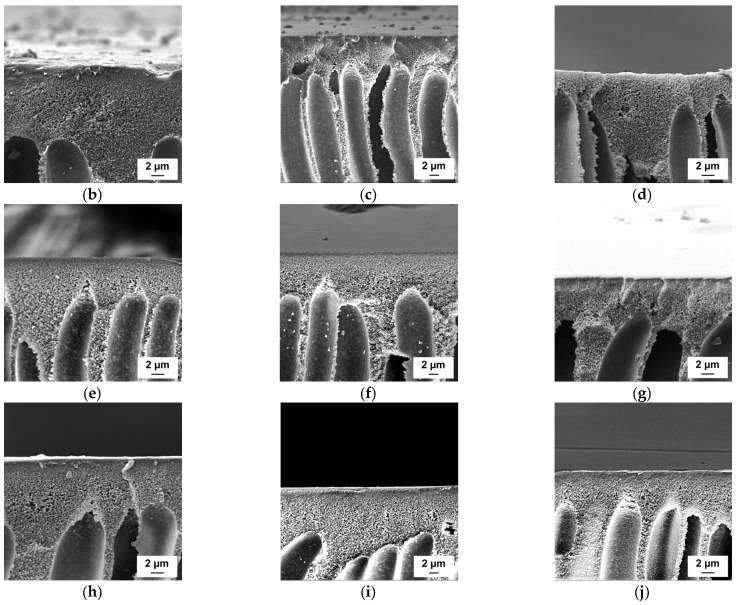
SEM micrographs of the cross-sections in the region near the skin layer of the reference PSF membrane (**a**) and membranes, modified by the introduction of PAA to the precipitation medium (**b**–**j**): (**a**) PA-0; (**b**) PA-100-1.0; (**c**) PA-250-1.0; (**d**) PA-450-1.0; (**e**) PA-100-1.5; (**f**) PA-250-1.5; (**g**) PA-450-1.5; (**h**) PA-100-2.0; (**i**) PA-250-2.0; and (**j**) PA-450-2.0.

**Figure 4:**
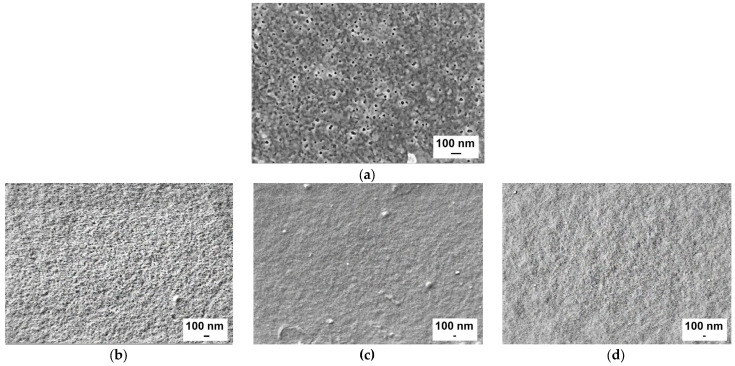

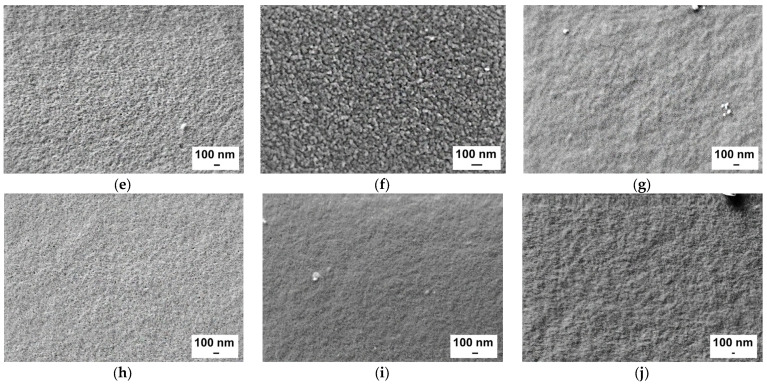
SEM micrographs of the membrane skin layer surfaces: (**a**) PA-0; (**b**) PA-100-1.0; (**c**) PA-250-1.0; (**d**) PA-450-1.0; (**e**) PA-100-1.5; (**f**) PA-250-1.5; (**g**) PA-450-1.5; (**h**) PA-100-2.0; (**i**) PA-250-2.0; and (**j**) PA-450-2.0.

The SEM images of the cross-sections, enlarged fragments of the cross-sections in the region near the skin layer and surfaces of the membrane skin layers are demonstrated in Figure 2, Figure 3 and Figure 4. Membranes developed within this study feature anisotropic structure with thin skin layer, transitional layer (just beneath the skin layer) and porous substructure permeated by big, elongated vacuoles. This structure usually originates from the instantaneous demixing mechanism during NIPS.

When a high-molecular-weight hydrophilic polymer or polyelectrolyte was introduced to the precipitation medium, the following effects were reported:

(1) Increase in the viscosity of coagulation medium;
(2) Decline in the rate of diffusion of solvent from the polymer film and coagulant inside the polymer film;
(3) Reduction in the speed of the formation of the membrane [47,48,49,50,51]. It was discussed previously that addition of polyelectrolyte to the coagulation medium mainly influences the kinetics of phase separation during NIPS and has no influence on thermodynamics [47,48,49,50,51].

It was observed that regardless of the molecular weight of PAA, the membrane matrix becomes denser and less porous when aqueous solutions of PAA are applied as coagulation medium. The vacuoles step away from the top of the membrane in comparison with the reference membrane PA-0 (Figure 2), and the thickness of both transitional layer and selective layer increases (Figure 2 and Figure 3). Overall, the introduction of PAA to the precipitation medium results in the formation of a more uniform structure of the membrane cross-section with thicker and strait vacuoles. Large pores near the bottom of the membrane become smaller or disappear. All these effects are attributed to the diminishing of the rate of the exchange “solvent-non-solvent” during phase separation; so, the formation of the membrane structure occurs in more equilibrium conditions.

Actually, no significant difference is observed between the cross-sections of the membranes obtained using PAA with different molecular weights (Figure 2). However, it was revealed that the transitional layer and selective layer feature denser structures and turn thicker when PAA molecular weight increases at all studied concentrations (Figure 3).

It was revealed that pore size and porosity significantly go down with a rise in PAA molecular weight and concentration (Figure 4). The pore size of membranes modified with PAA with molecular weights of 250,000 and 450,000 is so small that the pores cannot be detected at the studied magnification of the instrument. This trend is due to the rise in viscosity of precipitation media, which results in a decline in the rate of phase separation.

Thus, an increase in the molecular weight of PAA yields an increase in the thickness and density of the skin layer and transitional layer and a decrease in the pore size of the selective layer, which is attributed to the decline in the rate of “solvent-non-solvent” exchange due to the rise in the viscosity of the coagulation medium. 

### 3.3. Influence of the PAA Molecular Weight on the Topography of the Selective Layer Surface 

**Figure 5:**
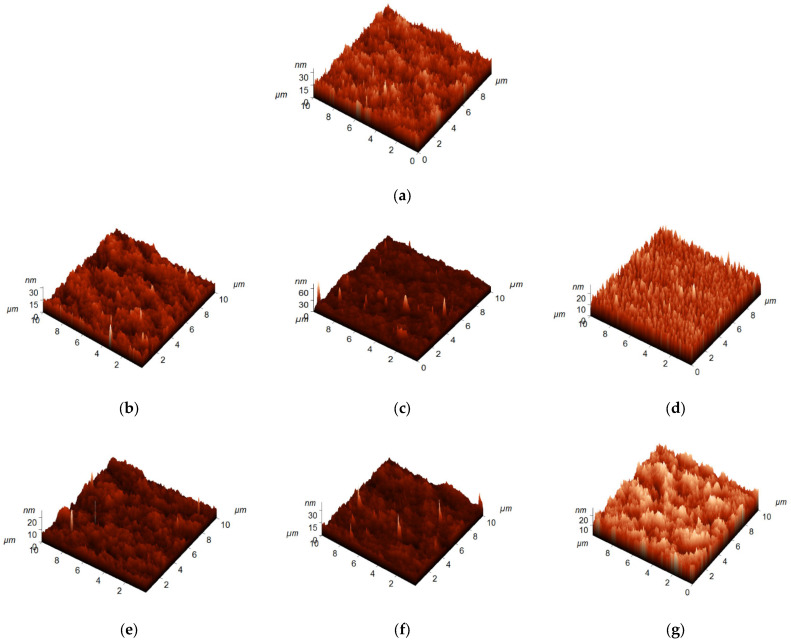

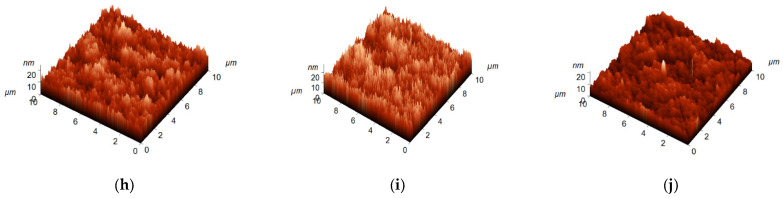
AFM images of the membrane skin layers: (**a**) PA-0; (**b**) PA-100-1.0; (**c**) PA-250-1.0; (**d**) PA-450-1.0; (**e**) PA-100-1.5; (**f**) PA-250-1.5; (**g**) PA-450-1.5; (**h**) PA-100-2.0; (**i**) PA-250-2.0; and (**j**) PA-450-2.0.

The topography of the surfaces of the PSF and PSF/PAA membrane selective layer was studied by AFM (Figure 5, Table 2). Surface roughness parameters (average roughness (R_a_) and root mean square surface roughness (R_q_)) were determined and are presented in Table 2. The surface of the skin layer of both reference and modified membranes features a typical structure which consists of polymer nodules usually formed in the NIPS process. Modification by PAA does not substantially change the surface topography. However, it was revealed that a rise in PAA molecular weight results in a slight increase in the surface roughness parameters (Table 2). For instance, membranes modified by PAA with a molecular weight of 450,000 g·mol^−1^ feature slightly higher R_a_ and R_q_ compared with both the PA-0 membrane and membranes modified with PAA with molecular weights of 100,000 and 250,000 g·mol^−1^. This can be due to the higher viscosity of coagulation bath and presence of PAA associates in the aqueous solution when PAA molecular weight increases up to 450,000 g·mol^−1^. The heterogeneity of the coagulation bath may result in the nonuniform diffusion of these associates to the nascent membrane during membrane preparation and an increase in membrane surface roughness.

However, it was found that surface roughness parameters change nonmonotonically for membranes of PA-100 and PA-250 series when PAA concentration in coagulation medium increases. When 1.0 wt.% of PAA is added to the coagulation medium surface, roughness parameters slightly increase compared with the reference membrane. When PAA concentration increases up to 1.5 wt.%, R_a_ and R_q_ decrease compared with both the reference membrane and PA-100-1.0 and PA-250-1.0 membranes. When PA concentration reaches 2.0 wt.%, R_a_ and R_q_ slightly rise in comparison with PA-100-1.5 an PA-250-1.5 membranes but remain lower compared with the reference membrane (PA-100-2.0) or become practically the same as the reference membrane (PA-250-2.0). This trend can be due to the combination of two different factors which have opposite effects on the surface roughness parameters: the pore size and porosity of the membrane selective layer and the heterogeneity and viscosity of the coagulation medium. A decrease in pore size and porosity can yield a smoother surface of the membrane selective layer, but in the case of PA-450-1.5 and PA-450-2.0 membranes, a decrease in the pore size and porosity is balanced by the increase in the heterogeneity of the coagulation medium; so, surface roughness parameters gradually increase with the rise in concentration of PAA with M_n_ = 450,000 g·mol^−1^ in coagulation bath. In the case of PAA with lower molecular weight, a decrease in the values of surface roughness parameters at PAA concentration of 1.5 wt.% is due to the decrease in pore size. Then, when PAA concentration increases up to 2.0 wt.%, the heterogeneity of the coagulation bath increases, which is overlapped with the decrease in pore size and porosity. As a result, surface roughness parameters for PA-100-2.0 and PA-250-2.0 membranes slightly increase compared with the PA-100-1.5 and PA-250-1.5 membranes. 

It is worth mentioning that, nevertheless, both reference and modified membranes have very smooth surface skin layers. 

### 3.4. Effect of the PAA Molecular Weight on the Water Contact Angle of Membrane Skin Layer

**Figure 6:**
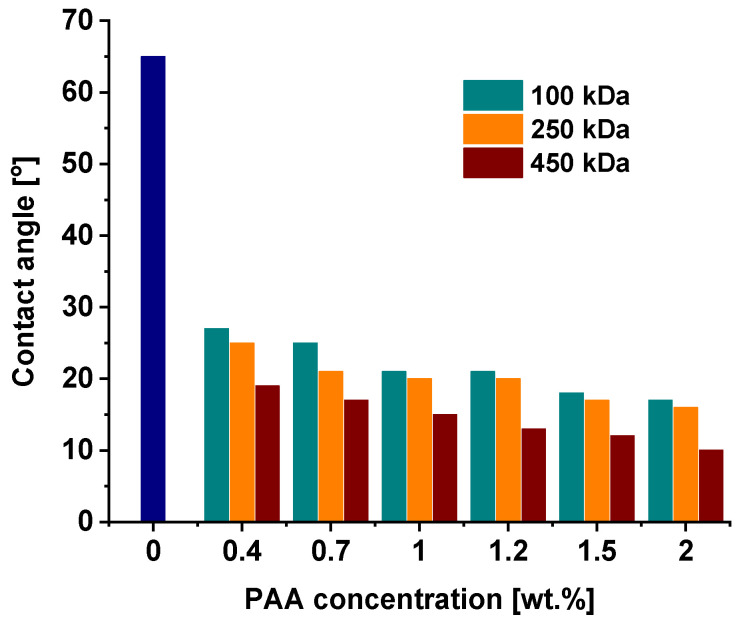
Dependence of the water contact angle of the membrane skin layer on the concentration of PAA of different molecular weights in the coagulation bath.

The introduction of PAA to the coagulation medium, increase in concentration and molecular weight of PAA lead to the dramatic hydrophilization of the membrane selective layer, which is proved by the decrease in water contact angle (Figure 6). For instance, it was found that the addition of 0.4 wt.% of PAA with a molecular weight of 100,000 g·mol^−1^ decreases the water contact angle from 65 ± 2° for the reference PSF membrane down to 27 ± 2° for PAA−100-0.4; 25 ± 2°for PAA-250-0.4; and down to 19 ± 2° for PAA-450-0.4. These data prove that the addition of even a small amount of PAA to coagulation medium is an efficient tool for the hydrophilization of membrane surfaces. When PAA concentration in a coagulation bath increases, the water contact angle continues falling down for all membranes, regardless of the molecular weight of PAA. Moreover, the higher the molecular weight of PAA, the more efficiently the hydrophilization of the membrane surface occurs due to the immobilization of a greater quantity of highly hydrophilic carboxylic groups, which is proved by FTIR spectra (Figure 1). When PAA concentration in a coagulation medium rises up to 2.0 wt.%, the water contact angle decreases down to 17 ± 2°, 16 ± 2° and 10 ± 2° for PAA-100-2.0, PAA-250-2.0 and PAA-450-2.0, respectively.

### 3.5. Effect of the PAA Molecular Weight on ξ-Potential of the Membrane Skin Layer

**Figure 7:**
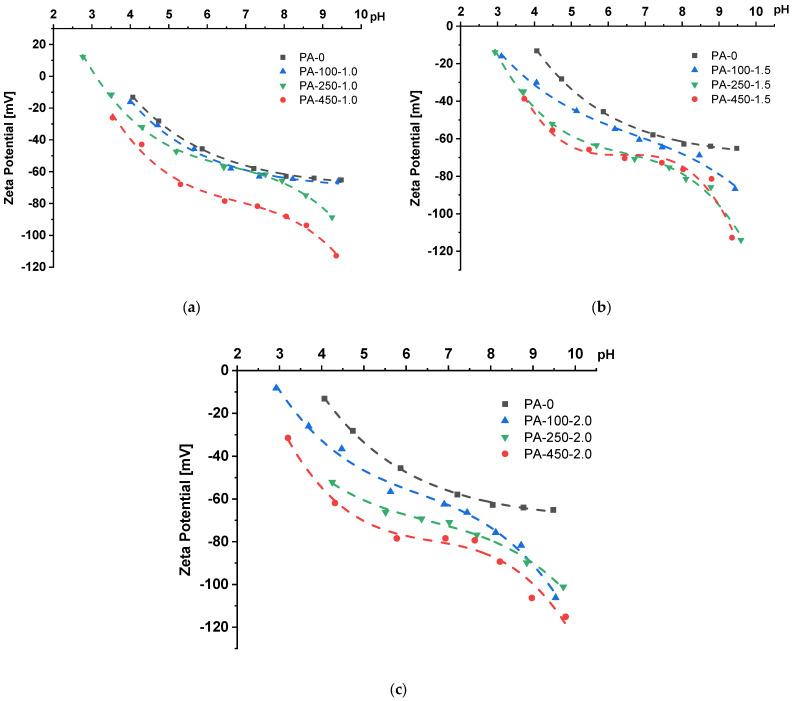
Dependence of ξ-potential of the surface of membrane skin layer on pH for PSF membranes obtained using additives of PAA of different concentrations, wt.%: (**a**) 1.0; (**b**) 1.5; (**c**) 2.0.

The addition of polyelectrolytes to the coagulation bath provides an opportunity to tailor the charge to the membrane surface during the membrane preparation in one step. The dependencies of zeta potential of the surface of the membrane selective layer on pH for membranes modified by the addition of PAA of different molecular weights and concentrations were obtained by the measurements of tangential flow streaming potential and are presented in Figure 7.

It was found that for both the reference and modified membranes, the ξ-potential of the surface of the membrane selective layer is negative for pH 3–10. A rise in pH leads to the decline in ξ-potential (the values become more negative), both for the reference PSF and PSF/PAA membranes. However, the mechanism for the decrease in zeta potential is different for the reference PSF and PSF/PAA membranes. In the first case, the mechanism is the adsorption of anions of electrolyte (Cl^−^) and hydroxyl ions on the membrane surface, and in the second case, it is due to the dissociation of carboxylic groups of PAA embedded in the selective layer [48,49]. The dissociation of carboxylic groups is known to occur at a pH higher than pK_a_ = 4.5.

Hence, an increase in PAA molecular weight and concentration has a significant impact on the values of ξ-potential due to the increase in the density of carboxylic groups on the surface of the membrane selective layer. For instance, the reference PSF membrane features a ξ-potential of −65 mV at pH = 9.5 and isoelectric point of 3.4. For all studied concentrations of PAA in the coagulation medium (1.0 wt.%, 1.5 wt.% and 2.0 wt.%), membranes can be arranged as follows according to the decrease in ξ-potential of the membrane selective layer:

PA − 100 > PA − 250 > PA − 450

For instance, at PAA concentration 2.0 wt.% in the coagulation bath, the modified membranes feature a ξ-potential of −76 mV, −101 mV and −106 mV for PA-100-2.0, PA-250-2.0 and PA-450-2.0, correspondingly. Moreover, zero fit suggests that membranes modified by PAA are characterized by an isoelectric point of around 2.5.

Thus, an increase in the molecular weight of PAA up to 450,000 g·mol^−1^ added to the coagulation medium during NIPS allows tailoring more negative charge to the membrane surface, which can enhance membrane antifouling performance, especially combined with extremely high hydrophilicity of the membrane surface discussed previously (Section 3.4).

### 3.6. Effect of PAA Molecular Weight and Concentration in the Coagulaton Bath on the Membrane Separation Performance

**Figure 8:**
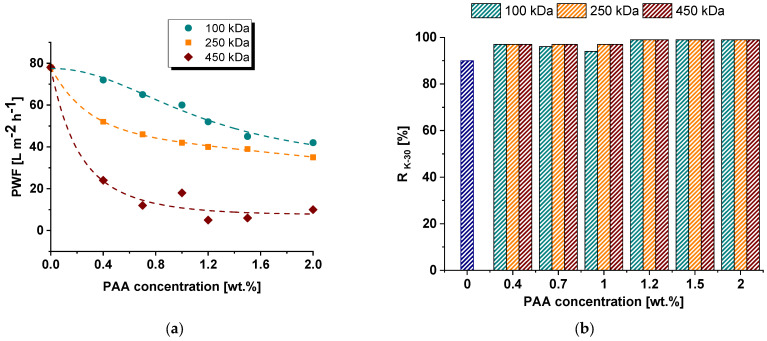
Pure water flux (**a**) and PVP K-30 rejection coefficient (R_K−30_) (**b**) versus PAA concentration in the coagulation bath.

**Figure 9:**
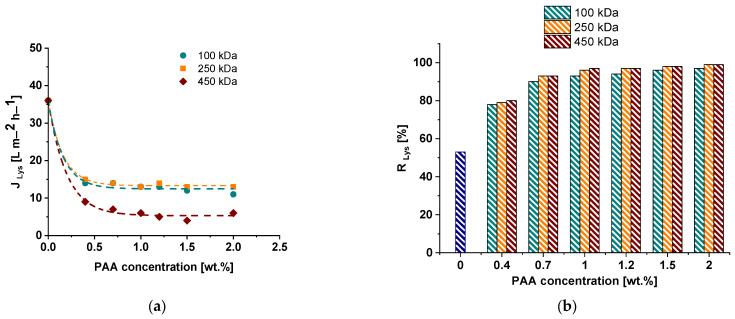
The dependence of 0.5 wt.% lysozyme solution flux (**a**) (J_Lys_) and lysozyme rejection coefficient (**b**) (R_Lys_) on the PAA concentration in the coagulation bath.

The membrane separation performance of the reference and modified membranes was investigated during an ultrafiltration of 0.5 wt.% lysozyme solution in phosphate buffer at pH = 7.2–7.4 and 0.3 wt.% PVP K−30 aqueous solution (Figure 8 and Figure 9). Pure water flux was found to substantially fall down when PAA was introduced to the coagulation medium. For instance, the PWF of the reference membrane was 78 L·m^−2^·h^−1^. When even 0.4 wt.% of PAA was added to the coagulation bath, the PWF decreases depending on PAA molecular weight: down to 72 L·m^−2^·h^−1^ for the PA-100-0.4 membrane, 52 L·m^−2^·h^−1^ for PA-250-0.4 membrane and 24 L·m^−2^·h^−1^ for PA-450-0.4 membrane. An increase in PAA concentration in the coagulation medium results in a further decline in PWF. The higher the molecular weight of PAA, the lower the membrane permeability at all studied PAA concentrations. When 2.0 wt.% of PAA is introduced to the coagulation medium, PWF decreases down to 40 L·m^−2^·h^−1^ for the PA-100-2.0 membrane, 35 L·m^−2^·h^−1^ for PA-250-2.0 and 10 L·m^−2^·h^−1^ for PA-450-2.0 membrane (Figure 8a). This trend is in good agreement with the change in membrane structure discussed in Section 3.2: the increase in PAA molecular weight as well as concentration results in the formation of a thicker and denser selective layer and a decrease in pore size and porosity of the selective layer (Figure 2, Figure 3 and Figure 4). 

It was revealed that the addition of PAA to the coagulation medium yields an increase in PVP K-30 rejection regardless of the molecular weight of PAA from 90% for the reference membrane for up to 97–99% for the membranes modified with PAA due to decrease in pore size of the selective layer (Figure 8b). Lysozyme solution flux also declines when PAA is introduced to coagulation medium and PAA concentration increases (Figure 9a). It was found that the addition of PAA with molecular weights of 100,000 and 250,000 g·mol^−1^ to the coagulation bath results in similar values of flux at practically all studied PAA concentrations. However, the lysozyme solution flux of membranes modified with PAA with 450,000 g·mol^−1^ is lower (Figure 9a). It was revealed that lysozyme rejection substantially increases from 53% for the reference PSF membrane to 78–80% for modified membranes when 0.4 wt.% PAA is added to coagulation bath (Figure 9b). This is attributed to the decrease in the pore size of the membrane selective layer (Figure 4). The increase in concentration and PAA molecular weight leads to the increase in lysozyme rejection, reaching 96–99% for membranes modified by the addition of more than 1 wt.% of PAA with molecular weights of 250,000 and 450,000 g·mol^−1^. It is worth noting that the lysozyme molecule and membrane surface are oppositely charged at pH = 7.2–7.4. Along with extremely high hydrophilicity, an opposite surface charge has to facilitate lysozyme penetration through the membrane. However, very small pore size combined with the possibility of interaction of lysozyme with embedded PAA chains which are stretched away from the membrane matrix yield efficient lysozyme rejection. So, a higher molecular weight of PAA yields longer macromolecular chains which extend out of the polymer matrix and are capable of interacting with lysozyme molecules via electrostatic interaction and formation of hydrogen bonds. This interaction increases lysozyme rejection. 

Thus, an increase in PAA molecular weight results in the decrease in membrane permeability and rise in lysozyme rejection.

### 3.7. Effect of the PAA Molecular Weight and Concentration in the Coagulatin Bath on the Membrane Antifouling Performance 

**Figure 10:**
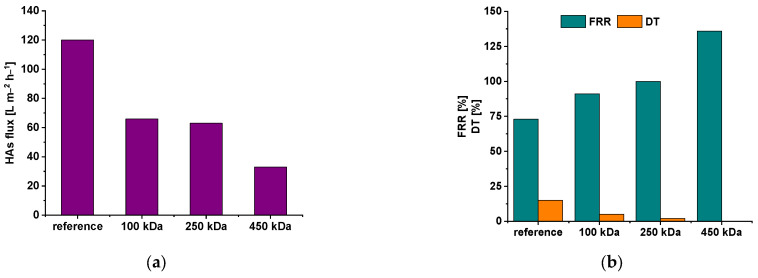
Dependence of 0.005 wt.% HAs solution flux (**a**), fouling recovery ratio (FRR, %) and total flux decline ratio (DT, %) (**b**) on the PAA molecular weight added to coagulation bath.

Membrane antifouling performance was investigated during ultrafiltration of HAs solution in tap water with subsequent cleaning by filtration of distilled water. PAA-100-1.5, PAA-250-1.5 and PA-450-1.5 membranes were selected for this experiment due to the combination of low water contact angle, high negative zeta potential at high pH and high rejection values. However, membrane pure water flux is higher for membranes modified by the addition of 1.5 wt.% PAA to coagulation medium compared with the membranes modified by the addition of 2.0 wt.% PAA. Membrane flux, fouling parameters, feed and permeate solution characteristics for the PSF reference and PSF/PAA membranes are presented in Figure 10 and Table 3.

It was established that HAs solution flux declines from 120 L·m^−2^·h^−1^ for the reference PSF membrane down to 66 L·m^−2^·h^−1^, 63 L·m^−2^·h^−1^ and 33 L·m^−2^·h^−1^ with the increase in PAA molecular weight for PA-100-1.5, PA-250-1.5 and PA-450-1.5, respectively (at 0.15 MPa) (Figure 10a). However, an increase in PAA molecular weight yields a significant increase in membrane fouling recovery ratio (FRR) and a decrease in the total flux decline ratio (DT) due to the higher negative surface ξ-potential, lower water contact angle and smaller pore size of the membranes modified by PAA with higher molecular weight (Figure 4, Figure 5 and Figure 6 and Figure 10b). It is worth noting that FRR is 100% and 136%, and DT is 2% and 0% for PA-250-1.5 and PA-450-1.5 membranes, respectively. An FRR value higher than 100% for PA-450-1.5 membrane maybe due to the changes in conformation of PAA chains which extend out from the polymer matrix of membrane. These changes in PAA conformation may result from the interactions with the components of the feed solution. 

Negative surface charge substantially contributes to the mitigation of membrane fouling due to the negative charge of HAs molecules at pH = 7.9 in the feed solution, which facilitates electrostatic repulsion between membrane surface and foulants [58]. Moreover, it was revealed that PSF/PAA membranes are more effective in the removal of color and iron from the feed HAs solution compared with the reference membrane (Table 3). The color of the feed solution mostly results from the dissolved HAs and iron compounds. Higher removal efficiency of PSF/PAA membranes is due to the high negative charge, which provides electrostatic adsorption of positively charged iron ions and rejection of negatively charged HAs molecules. An increase in PAA molecular weight was shown to decrease iron content in permeate due to the higher density of carboxylic groups and higher negative ξ-potential of the membrane selective layer surface (Table 3).

## 4. Conclusions

A systematic study of the influence of the molecular weight of PAA (100,000, 250,000 and 450,000 g·mol^−1^) added to precipitation medium during polysulfone membrane preparation via non-solvent-induced phase separation was carried out. It was established that an increase in PAA molecular weight from 100,000 g·mol^−1^ to 450,000 g·mol^−1^ had a significant effect on membrane pore structure, hydrophilic–hydrophobic balance, surface roughness and ξ-potential. Membranes obtained via the addition of PAA with higher molecular weight demonstrated smaller pore size and porosity, extremely high hydrophilicity and higher values of negative charge of the membrane surface. It was revealed that with the increase in PAA molecular weight from 100,000 to 450,000 g·mol^−1^ (at 2.0 wt.% PAA in the coagulation bath), water contact angle decreased from 17 ± 2° to 10 ± 2°, which is a dramatic drop compared with the reference membrane (65 ± 2°). It was shown that the increase in PAA concentration from 0.4 wt.% to 2.0 wt.% for all studied PAA molecular weights yielded a substantial decrease in water contact angle (from 27 ± 2° to 17 ± 2° for PAA with M_n_ = 100,000 g·mol^−1^; from 25 ± 2° to 16 ± 2° for PAA with M_n_ = 250,000 g·mol^−1^; from 19 ± 2° to 10 ± 2° for PAA with M_n_ = 450,000 g·mol^−1^). It was revealed that an increase in PAA molecular weight and concentrations in the coagulation medium led to an increase in negative values of ξ-potential. An increase in PAA molecular weight and concentration led to a decrease in membrane permeability, an increase in rejection and tailoring excellent antifouling performance in the ultrafiltration of humic acid solutions. For instance, fouling recovery ratio increased from 73% for the reference membrane up to 91%, 100% and 136% for membranes modified with the addition to the coagulation bath of 1.5 wt.% of PAA with molecular weights of 100,000 g·mol^−1^, 250,000 g·mol^−1^ and 450,000 g·mol^−1^, respectively.

Overall, the addition of PAA of different molecular weights to the precipitation medium is an efficient tool to adjust membrane separation and antifouling properties for different separation tasks.

## Figures and Tables

**Table 1:** Abbreviations of PSF ultrafiltration membranes obtained using PAA aqueous solutions as coagulation bath.

Abbreviation	PAA Molecular Weight, g·mol^−1^	PAA Concentration, wt.%
PA-0	-	0
PA-100–0.4	100,000	0.4
PA-100–0.7		0.7
PA-100–1.0		1.0
PA-100–1.2		1.2
PA-100–1.5		1.5
PA-100–2.0		2.0
PA-250–0.4	250,000	0.4
PA-250–0.7		0.7
PA-250–1.0		1.0
PA-250–1.2		1.2
PA-250–1.5		1.5
PA-250–2.0		2.0
PA-450–0.4	450,000	0.4
PA-450–0.7		0.7
PA-450–1.0		1.0
PA-450–1.2		1.2
PA-450–1.5		1.5
PA-450–2.0		2.0

**Table 2:** Surface roughness parameters of the membrane skin layers.

Coagulation Medium	M_n_ = 100,000 g·mol^−1^		M_n_ = 250,000 g·mol^−1^		M_n_ = 450,000 g·mol^−1^	
	Roughness Parameters					
	R_a_, nm	R_q_, nm	R_a_, nm	R_q_, nm	R_a_, nm	R_q_, nm
Water	2.7	3.3	2.7	3.3	2.7	3.3
1.0 wt.% PAA	2.3	2.9	2.8	3.7	3.0	3.7
1.5 wt.% PAA	1.5	1.9	1.8	2.4	2.9	3.7
2.0 wt.% PAA	2.1	2.7	2.8	3.5	3.5	3.9

**Table 3:** Permeate parameters during ultrafiltration of 0.005% HAs solution using developed membranes.

Membrane Designation	Permeate Parameters		
	Color (λ = 400 nm)	pH	c (Fe) [mg L^−1^]
Feed solution			
	0.56	7.9	270
**Permeate solution**			
PA-0	0.13	7.9	2.7
PA-100-1.5	0.01	7.9	0.4
PA-250-1.5	0.01	7.9	0.3
PA-450-1.5	0.01	7.9	0.1

## Data Availability

Data presented in this study are available on request from the corresponding author.

## Supplementary Materials

The following supporting information can be downloaded at: https://www.mdpi.com/article/10.3390/polym15071664/s1, Figure S1: FTIR spectra of PSF and PSF/PAA membranes depending on the concentration of PAA in the coagulation bath, PAA molecular weight, g·mol^−1^: a—100,000; b—250,000; c—450,000.
