# Supplementary material for: Effect of the Addition of Polyacrylic Acid of Different Molecular Weights to Coagulation Bath on the Structure and Performance of Polysulfone Ultrafiltration Membranes
Source: Polymers (Basel). 2023 Mar 27;15(7):1664. doi: 10.3390/polym15071664 (PMC10097043; doi:10.3390/polym15071664)
Supplement: Supplementary file 1 [file polymers-15-01664-s001.zip › polymers-2294066-supplementary.pdf]

Supplementary materials

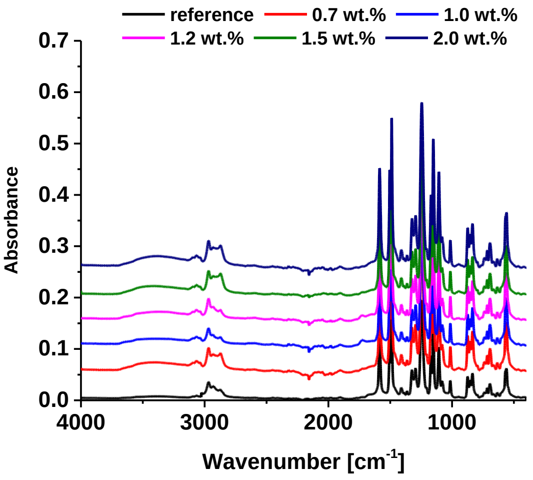

(a)

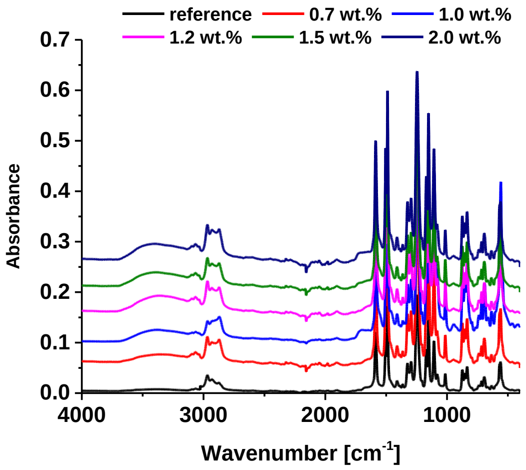

(b)

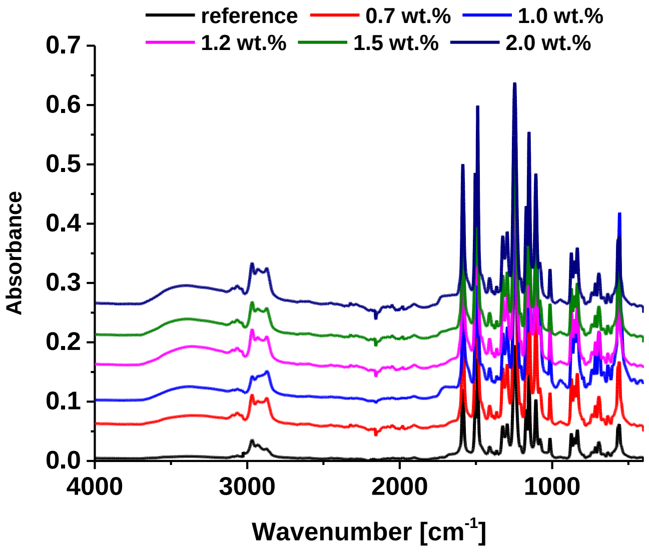

(c)

Figure S1. FTIR spectra of PSF and PSF/PAA membranes depending on the concentration of PAA in the coagulation bath, PAA molecular weight, g·mol^{-1}: a – 100,000; b – 250,000; c – 450,000.
